# Incorporating measurement error in *n* = 1 psychological autoregressive modeling

**DOI:** 10.3389/fpsyg.2015.01038

**Published:** 2015-07-28

**Authors:** Noémi K. Schuurman, Jan H. Houtveen, Ellen L. Hamaker

**Affiliations:** ^1^Department of Methodology and Statistics, Utrecht UniversityUtrecht, Netherlands; ^2^Academic Centre of Psychiatry, Groningen UniversityGroningen, Netherlands

**Keywords:** autoregressive modeling, *n* = 1, measurement error, Bayesian modeling, idiographic, time series analysis

## Abstract

Measurement error is omnipresent in psychological data. However, the vast majority of applications of autoregressive time series analyses in psychology do not take measurement error into account. Disregarding measurement error when it is present in the data results in a bias of the autoregressive parameters. We discuss two models that take measurement error into account: An autoregressive model with a white noise term (AR+WN), and an autoregressive moving average (ARMA) model. In a simulation study we compare the parameter recovery performance of these models, and compare this performance for both a Bayesian and frequentist approach. We find that overall, the AR+WN model performs better. Furthermore, we find that for realistic (i.e., small) sample sizes, psychological research would benefit from a Bayesian approach in fitting these models. Finally, we illustrate the effect of disregarding measurement error in an AR(1) model by means of an empirical application on mood data in women. We find that, depending on the person, approximately 30–50% of the total variance was due to measurement error, and that disregarding this measurement error results in a substantial underestimation of the autoregressive parameters.

## 1. Introduction

The dynamic modeling of processes at the within-person level is becoming more and more popular in psychology. The reason for this seems to be the realization that inter-individual differences, in many cases, are not equal to intra-individual differences. Indeed, studies that compare interindividual differences and intraindividual differences usually do not harbor the same results, exemplifying that conclusions based on studies of group averages (including cross-sectional studies and panel data studies), cannot simply be generalized to individuals (Nezlek and Gable, 2001; Borsboom et al., 2003; Molenaar, 2004; Rovine and Walls, 2006; Kievit et al., 2011; Madhyastha et al., 2011; Ferrer et al., 2012; Hamaker, 2012; Wang et al., 2012; Adolf et al., 2015).

The increased interest in analyses at the within-person level, and the increasing availability of technology for collecting these data, has resulted in an increase in psychological studies that collect intensive longitudinal data, consisting of many (say 25 or more) repeated measures from one or more individuals. A popular way to analyze these data currently is by autoregressive time series (AR) modeling, either by modeling the repeated measures for a single individual using classical *n* = 1 AR models, or by using multilevel extensions of these models, with the repeated measures for each individual modeled at level 1, and individual differences modeled at level 2 (Cohn and Tronick, 1989; Suls et al., 1998; Nezlek and Gable, 2001; Nezlek and Allen, 2006; Rovine and Walls, 2006; Moberly and Watkins, 2008; Kuppens et al., 2010; Lodewyckx et al., 2011; Madhyastha et al., 2011; Wang et al., 2012; De Haan-Rietdijk et al., 2014). In an AR model of order 1 [i.e., an AR(1) model], a variable is regressed on a lagged version of itself, such that the regression parameter reflects the association between this variable and itself at the previous measurement occasion (c.f., Hamilton, 1994; Chatfield, 2004). The reason for the popularity of this model may be the natural interpretation of the resulting AR parameter as inertia, that is, resistance to change (Suls et al., 1998). Resistance to change is a concept which is considered to be relevant to many psychological constructs and processes, including attention, mood and the development of mood disorders, and the revision of impressions and opinions (Geller and Pitz, 1968; Goodwin, 1971; Suls et al., 1998; Kirkham et al., 2003; Kuppens et al., 2010; Koval et al., 2012).

However, a problem with the regular AR(1) model is that it does not account for any measurement errors present in the data. Although AR models incorporate residuals, which are referred to as “innovations” or “dynamic errors,” these residuals are to be distinguished from measurement error. Simply put, the distinction between dynamic errors and measurement errors is that dynamic errors carry over to next measurement occasions through the autoregressive relationship, while measurement errors are specific to one measurement occasion. Therefore, even though taking measurement errors into account is considered business as usual in many psychological studies of interindividual differences, it is largely neglected in AR modeling. Two exceptions are formed by Wagenmakers (2004) and Gilden (2001)^1^, both of which concern studies on reaction time and accuracy in series of cognitive tasks. Gilden notes that there is evidence that some variance in reaction time is random (measurement) error as a result of key-pressing in computer tasks. Measurement error however is not limited to “accidentally” pressing the wrong button or crossing the wrong answer, but is made up of the sum of all the influences of unobserved factors on the current observation, that do not carry-over to the next measurement occasion. Disregarding measurement error distorts the estimation of the effects of interest (Staudenmayer and Buonaccorsi, 2005). This is quite problematic, considering that in psychological studies it is often impossible to directly observe the variable of interest, and it therefore seems likely (and this seems generally accepted among psychological researchers) that psychological research in general is prone to having noisy data.

The aim of this study is therefore three-fold. First, we aim to emphasize the importance of considering measurement error in addition to dynamic error in intensive longitudinal studies, and illustrate the effects of disregarding it in the case of the *n* = 1 autoregressive model. Second, we aim to compare two modeling strategies for incorporating measurement errors: (1) fitting an autoregressive model that includes a white noise term (AR+WN), and (2) fitting an autoregressive moving average (ARMA) model. These modeling strategies are the two most frequently suggested in the literature (e.g., in mathematical statistics, control engineering, and econometrics, c.f., Granger and Morris, 1976; Deistler, 1986; Chanda, 1996; Swamy et al., 2003; Staudenmayer and Buonaccorsi, 2005; Chong et al., 2006; Costa and Alpuim, 2010; Patriota et al., 2010). Third, our aim is to compare the performance of these models for a frequentist and a Bayesian estimation procedure. Specifically, for the frequentist procedure we will focus on a Maximum Likelihood (ML) procedure based on the state-space modeling framework, which is a convenient modeling framework for psychological longitudinal modeling, as it readily deals with missing data, and is easily extended to multivariate settings, or to include latent variables (Harvey, 1989). The Bayesian alternative shares these qualities, and has the additional advantage that the performance of the estimation procedure is not dependent on large samples (Dunson, 2001; Lee and Wagenmakers, 2005), while the performance of the frequentist ML procedure depends on asymptotic approximations, and in general requires large samples. This is convenient for the modeling of intensive longitudinal data, given that large amounts of repeated measures are often difficult to obtain in psychological studies. By means of a simulation study we will evaluate the parameter recovery performance of the Bayesian procedure for the ARMA(1,1) and the AR+WN model, and compare it to the ML procedure.

This paper is organized as follows. We start by introducing the AR(1) model, ARMA(1,1) model, and the AR(1)+WN model, and discussing their connections. After that, we present the methods for the simulation study, followed by the results. We present an empirical application concerning the daily mood of eight women, in order to further illustrate the consequences of disregarding measurement error in practice, and we end with a discussion.

## 2. Models

In this section we present the AR(1) model, and explain the difference between the dynamic errors that are incorporated in the AR(1) model, and measurement errors. After that we will introduce models that incorporate measurement errors, namely the autoregressive model with an added white noise term (AR(1)+WN model), and the autoregressive moving average (ARMA) model.

### 2.1. The AR(1) model

In order to fit an AR model, a large number of repeated measures is taken from one individual. Each observation, or score, *y*_*t*_ in the AR model consists of a stable trait part—the mean of the process denoted as μ, and a state part *ỹ*_*t*_ that reflects the divergence from that mean at each occasion. In an AR model of order 1, the state of the individual at a specific occasion *ỹ*_*t*_ depends on the previous state *ỹ*_*t* − 1_, and this dependency is modeled with the AR parameter ϕ. Specifically, the AR(1) model can be specified as

(1) $$\begin{array}{rcl} y_{t} & = & \mu +{ỹ}_{t}\\ {ỹ}_{t} & = & \phi {ỹ}_{t-1}+\epsilon_{t} \end{array}$$

(2) $$\begin{array}{rcl} \epsilon_{t} & \sim & N\left(0,\sigma_{\epsilon }^{2}\right). \end{array}$$

**Figure 1:**
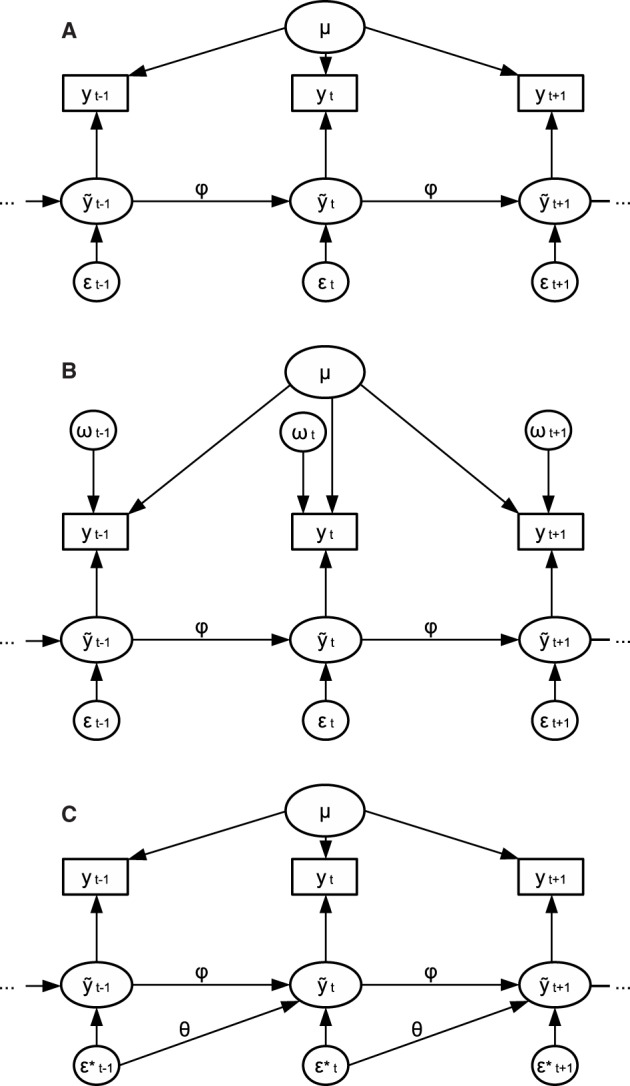
**(A)** Graphical representation of an AR(1) model. **(B)** Graphical representation of an AR(1)+WN model. **(C)** Graphical representation of an ARMA(1,1) model.

For a graphical representation of the model, see Figure 1A. A positive value for ϕ indicates that the score at the current occasion will be similar to that at the previous occasion— and the higher the positive value for ϕ, the more similar the scores will be. Therefore, a positive AR parameter reflects the inertia, or resistance to change, of a process (Suls et al., 1998). A positive AR parameter could be expected for many psychological processes, such as that of mood, attitudes, and (symptoms of) psychological disorders. A negative ϕ indicates that if an individual has a high score at one occasion, the score at the next occasion is likely to be low, and vice versa. A negative AR parameter may be expected for instance in processes that concern intake, such as drinking alcoholic beverages: If an individual drinks a lot at one occasion, that person may be more likely to cut back on alcohol the next occasion, and the following occasion drink a lot again, and so on Rovine and Walls (2006). An AR parameter close to zero indicates that a score on the previous occasion does not predict the score on the next occasion. Throughout this paper we consider stationary models, which implies that the mean and variance of *y* are stable over time, and ϕ lies in the range from −1 to 1 (Hamilton, 1994). The innovations ϵ_*t*_ reflect that component of each state score *ỹ*_*t*_ that is unpredictable from the previous observation. The innovations ϵ_*t*_ are assumed to be normally distributed with a mean of zero and variance $\sigma_{\epsilon }^{2}$.

### 2.2. Dynamic errors vs. measurement errors

The innovations ϵ_*t*_ perturb the system and change its course over time. Each innovations is the result of all unobserved events that impact the variable of interest at the current measurement occasion, of which the impact is carried over through the AR effect to the next few measurement occasions. Take for example hourly measurements of concentration: Unobserved events such as eating a healthy breakfast, a good night sleep the previous night, or a pleasant commute, may impact concentration in the morning, resulting in a heightened concentrating at that measurement occasion. This heightened concentration may then linger for the next few measurement occasions as a result of an AR effect. In other words, the innovations ϵ_*t*_ are “passed along” to future time points via ϕ, as can be seen from Figure 1A, and this is why they are also referred to as “dynamic errors.”

Measurement errors, on the other hand, do not carry over to next measurement occasions, and their effects are therefore restricted to a single time point. This can also be seen from Figure 1B: The dynamic errors are passed from *y*_*t* − 1_ to *y*_*t*_ through the AR effect while the measurement errors ω_*t*_ are specific to each observation. Classical examples of measurement error, which are moment-specific, are making an error while filling in a questionnaire, or accidentally pressing a (wrong) button during an experiment (e.g., Gilden, 2001). However, any unobserved effect of which the influence is not carried over to the next measurement occasion may also be considered as measurement error, rather than dynamic error. The only distinguishing characteristic of measurement errors and dynamic errors is that the latter's influence lingers for multiple measurement occasions. Therefore, in practice, what unobserved effects will end up as measurement error, and what effects will end up as dynamic error, will depend largely on the measurement design of the study, such as on the frequency of the repeated measures that are taken. For example, some unobserved effects may carry-over from minute to minute (e.g., having a snack, listening to a song), but not from day to day—if measurements are then taken every minute, these unobserved effects will end up in the dynamic error term, but if measurements are taken daily, such effects will end up in the measurement error term. As such, the more infrequent measurements are taken, the more measurement errors one can expect to be present in the data, relative to the dynamic errors.

In psychological research measurement is complicated, and likely to be noisy. As such, the contribution of measurement error variance to the total variance of the measured process may be considerable. Ignoring this contribution will result in biased parameter estimates. Staudenmayer and Buonaccorsi (2005) have shown that in the case of an AR(1) model, ϕ will be biased toward zero. Specifically, the estimated AR coefficient $\hat{\phi }$ will be equal to (1 − λ) * ϕ, where ϕ is the true AR parameter and λ is the proportion of measurement error variance to the total variance. Hence, in order to prevent the measurement error from biasing estimates of ϕ, it is necessary to take measurement error into account in the modeling procedure. This approach has two advantages: First, it leads to less biased estimates of ϕ, and second, it allows us to investigate to what extent the measurements are determined by measurement error.

### 2.3. Incorporating measurement error: the AR(1)+WN model

A relatively simple way to incorporate measurement error in dynamic modeling is to add a noise term to the model, typically white noise, to represent the measurement error. White noise is a series of random variables that are identically and independently distributed (Chatfield, 2004). For the AR model with measurement error (AR(1)+WN), the white noise ω_*t*_ is simply added to each observation *y*_*t*_ (see Figure 1B). We assume that this white noise is normally distributed with a mean of zero and variance $\sigma_{\omega }^{2}$. This results in the following model specification for the AR(1)+WN model

(3) $$\begin{array}{rcl} y_{t} & = & \mu +{ỹ}_{t}+\omega_{t}\\ {ỹ}_{t} & = & \phi {ỹ}_{t-1}+\epsilon_{t} \end{array}$$

(4) $$\begin{array}{rcl} \epsilon_{t} & \sim & N\left(0,\sigma_{\epsilon }^{2}\right) \end{array}$$

(5) $$\begin{array}{rcl} \omega_{t} & \sim & N\left(0,\sigma_{\omega }^{2}\right). \end{array}$$

Important to note is that when ϕ is equal to zero, the measurement error and dynamic error will no longer be discernible from each other, because they are only discernible from each other from the merit that the innovations are passed to future time points through ϕ, while the measurement errors are not. In that case, the AR(1)+WN model is no longer identified, which is problematic for estimating the model parameters. Further note that when ϕ is nonzero, the higher |ϕ|, the easier it will be to discern measurement error from the innovations, and as such the model will be easier to identify empirically, and likely easier to estimate. Hence, in this sense the (empirical) identification of the AR(1)+WN model may be seen as dimensional rather than dichotomous, ranging from unidentified when ϕ is zero, to maximally empirically identified when |ϕ| is one.

### 2.4. Incorporating measurement error: the ARMA(1,1) model

Another way to incorporate measurement error into an AR(1) model that is relatively frequently suggested in the literature on dynamic modeling with measurement error, is to use an autoregressive moving average (ARMA) model (see for instance: Granger and Morris, 1976; Deistler, 1986; Chanda, 1996; Swamy et al., 2003; Wagenmakers et al., 2004; Staudenmayer and Buonaccorsi, 2005; Costa and Alpuim, 2010; Patriota et al., 2010). Granger and Morris (1976) have shown that the AR(p)+WN model is equivalent to an ARMA(p,p) model, where p stands for the number of lags included in the model. As a result, an ARMA(1,1) model can be used as an indirect way to fit an AR(1) model and take measurement error into account (Granger and Morris, 1976; Staudenmayer and Buonaccorsi, 2005; Wagenmakers et al., 2004). One advantage of fitting an ARMA(1,1) model rather than fitting an AR(1)+WN model directly, is that it can be estimated with a wide range of estimation procedures, and a wide range of software, including for instance SPSS. A second important advantage is that the ARMA(1,1) is identified when the value of ϕ is equal to zero, so that in practice it may be easier to estimate than the AR(1)+WN model.

An ARMA(1,1) process consists of an AR(1) process, and a moving average process of order 1 [MA(1)]. In an MA(1) process, the current state *ỹ*_*t*_ depends not only on the innovation, $\epsilon_{t}^{*}$, but also on the previous innovation $\epsilon_{t-1}^{*}$, through moving average parameters θ.^2^ For example, consider the daily introverted behavior for a specific person. On a certain day, the person has a shameful experience, resulting in a strong boost (e.g., an innovation or perturbation) in introverted behavior. The next day, this person may display lingering heightened introverted behavior from the previous day as a result of an AR effect, but there may also be a delayed response to the perturbation from yesterday, for instance because the person remembers the events of the previous day. The strength of the delayed response depends on the size of θ. The ARMA(1,1) model, which is depicted in Figure 1C, can be specified as:

(6) $$\begin{array}{rcl} y_{t} & = & \mu +{ỹ}_{t}\\ {ỹ}_{t} & = & \phi {ỹ}_{t-1}+\theta \epsilon_{t-1}^{*}+\epsilon_{t}^{*} \end{array}$$

(7) $$\begin{array}{rcl} \epsilon_{t}^{*} & \sim & N\left(0,\sigma_{\epsilon }^{2*}\right). \end{array}$$

The ARMA(1,1) model is characterized by four parameters, that is, the mean μ, AR parameter ϕ, moving average parameter θ, and innovation variance $\sigma_{\epsilon }^{2*}$. The model is stationary when ϕ lies between –1 and 1, and is invertible if θ lies between −1 and 1 (Chatfield, 2004; Hamilton, 1994).

If the true underlying model is an AR(1)+WN model, the ϕ and μ parameter in an ARMA(1,1) will be equal to those of the AR(1)+WN model. Granger and Morris (1976) have shown that the innovation variance $\sigma_{\epsilon }^{2}$ and measurement error variance $\sigma_{\omega }^{2}$ can be calculated from the estimated θ, ϕ, and $\sigma_{\epsilon }^{2*}$ as follows (see also Staudenmayer and Buonaccorsi, 2005),

(8) $$\begin{array}{rcl} \sigma_{\omega }^{2} & = & {\left(-\phi \right)}^{-1}\theta \sigma_{\epsilon }^{2*}, \end{array}$$

(9) $$\begin{array}{rcl} \sigma_{\epsilon }^{2} & = & \left(1+\theta^{2}\right)\sigma_{\epsilon }^{2*}-\left(1+\phi^{2}\right)\sigma_{\omega }^{2}. \end{array}$$

It is important to note that while the AR(1)+WN models is equivalent to an ARMA(1,1) model, an ARMA(1,1) models is not necessarily equivalent to an AR(1)+WN model. That is, it is only possible to transform the ARMA(1,1) parameters to AR(1)+WN model parameters under these restrictions in line with an underlying AR(1)+WN model (Granger and Morris, 1976; Staudenmayer and Buonaccorsi, 2005):

(10) $$\begin{array}{l} \frac{1}{1+\phi^{2}}>\frac{\theta }{1+\theta^{2}}\left(-\phi^{-1}\right)\geq 0 \end{array}$$

## 3. Simulation study methods

We present a simulation study in which we simulate data according to an AR process with added measurement error. We fit an AR(1) model to the data in order to illustrate the effects of ignoring any present measurement error, and compare the performance of the AR(1) model to the AR(1)+WN, and ARMA(1,1) model, which both account for measurement error. Furthermore, we will compare the performance of the Bayesian and frequentist estimation of these models.

### 3.1. Frequentist estimation

For the frequentist estimation of the AR(1) model and the ARMA(1,1) model a relatively wide range of procedures and software is available. Potential estimation procedures for fitting the AR(1)+WN model include specially modified Yule-Walker equations, and modified Least Squares estimation procedures (Chanda, 1996; Staudenmayer and Buonaccorsi, 2005; Dedecker et al., 2011). However, we opt to use the (linear, Gaussian) state-space model, for which the Kalman Filter (Harvey, 1989; Kim and Nelson, 1999) is used to estimate the latent states, while Maximum Likelihood is used to estimate the model parameters (c.f., Staudenmayer and Buonaccorsi, 2005, for this approach, but with the measurement error variance considered as known). This is an especially convenient modeling framework for psychological longitudinal modeling, as it readily deals with missing data, and is easily extended to multivariate settings, or to include latent variables (c.f., Hamilton, 1994; Harvey, 1989; Kim and Nelson, 1999).

In the state-space model representation, a vector of observed variables is linked to a vector of latent variables—also referred to as “state variables”—in the *measurement equation*, and the dynamic process of the latent variables is described through a first-order difference equation in the *state equation* (Hamilton, 1994; Harvey, 1989; Kim and Nelson, 1999). That is, the measurement equation is

(11) $$\begin{array}{l} y_{t}=d+F{\tilde{y}}_{t}+\omega_{t}\\ \omega_{t}\sim MvN\left(0,\;\Sigma_{\omega }\right), \end{array}$$

where ***y***_*t*_ is an *m*×1 vector of observed outcome variables, ***ỹ***_*t*_ is an *r*×1 vector of latent variables, ***d*** is an *m*×1 vector with intercepts for the observed variables, ***F*** is an *m*×*r* matrix of factor loadings, and **ω**_*t*_ is an *m*×1 vector of residuals that are assumed to be multivariate normally distributed with zero means and *m*×*m* covariance matrix **Σ**_ω_. The state equation (also referred to as the transition equation) is specified as

(12) $$\begin{array}{l} {\tilde{y}}_{t}=c+A{\tilde{y}}_{t-1}+\epsilon_{t}\\ \epsilon_{t}\sim MvN\left(0,\;\Sigma_{\epsilon }\right), \end{array}$$

where ***c*** is an *r*×1 vector of intercepts for the latent variables, ***A*** is an *r*×*r* matrix of structural coefficients, and **ϵ**_*t*_ is an *r*×1 vector of residuals, which are assumed to be multivariate normally distributed with zero means and *r*×*r* covariance matrix **Σ**_ϵ_.

The previously discussed AR(1) and AR(1)+WN model are both already specified in terms of a state-space representation in Equations (1) through (5) (simplified where possible). For the state-space model specification for the ARMA(1,1) model vector ***d*** is μ, ***F*** is $\left[\begin{array}{cc} 1 & 0 \end{array}\right]$^*T*^, ***ỹ***_*t*_ is ${\left[\begin{array}{cc} {\tilde{y1}}_{t} & {\tilde{y2}}_{t} \end{array}\right]}^{T}$, **Σ**_ω_ is a zero matrix, ***c*** is a zero vector, ***A*** is 2 × 2 matrix $\left[\begin{array}{cc} \phi & 0\\ 1 & 0 \end{array}\right]$, and 2 × 2 matrix **Σ**_ϵ_ is equal to ***H*^*T*^*H*** with ***H*** equal to $\left[\begin{array}{cc} \sigma_{1\epsilon^{*}} & \theta \sigma_{1\epsilon^{*}} \end{array}\right]$, where superscript *T* indicates the transpose.

To fit the frequentist state-space models we use R, with R packages FKF (Kalman Filter; Luethi et al., 2010) combined with R base package optim (for maximum likelihood optimization; R Development Core Team, 2012). Within optim we used optimization method l-bfgs-b, with lower bounds and upper bounds for ϕ and θ of −1 and 1, -Inf and Inf for μ, and 0 and Inf for $\sigma_{\epsilon }^{2}$, $\sigma_{\omega }^{2}$, and $\sigma_{\nu }^{2}$.

### 3.2. Bayesian estimation

Bayesian modeling shares a lot of conveniences with the frequentist state-space modeling framework: For instance, like frequentist state-space modeling procedures, Bayesian modeling can deal conveniently with missing data, is flexible in modeling multivariate processes, and in including latent variables in the model. Particular to Bayesian modeling is the relative ease in extending models to a hierarchical or multilevel setting (e.g., Lodewyckx et al., 2011; De Haan-Rietdijk et al., 2014). Another advantage may be the possibility to include prior information in the analysis, based, for instance, on expert knowledge or results from previous research (e.g., Rietbergen et al., 2011, 2014). Finally, the Bayesian estimation procedures are not dependent on large sample asymptotics like the frequentist procedures, and may therefore perform better for smaller samples (Dunson, 2001; Lee and Wagenmakers, 2005). Because currently there is no literature on the Bayesian estimation performance for the AR(1)+WN model, we will compare the performance of the Bayesian AR(1), ARMA(1,1), and AR(1)+WN model with the frequentist modeling equivalents in a simulation study.

In Bayesian estimation the information in the data, provided through the likelihood, is combined with a prior distribution using Bayes' rule (c.f., Gelman et al., 2003; Hoijtink et al., 2008). The prior distribution is specified such that it contains prior information the researcher would like to include in the analysis. Here we prefer to specify uninformative prior distributions that contain minimal prior information, such that their influence is minimal. Specifically, we use the following prior specifications across the three models: A *uniform*(0, 20) prior on $\sigma_{\omega }^{2}$, $\sigma_{\epsilon }^{2}$, and $\sigma_{\nu }^{2}$, a *uniform*(−1, 1) prior on ϕ and θ, and a *normal*(0, 0.001) prior for μ (specified with precision rather than variance). When the prior distribution and the likelihood are combined using Bayes' rule, this results in the posterior probability distribution or density of the estimated parameters. Summary statistics based on this distribution can then be used to summarize the information on the estimated parameters, for instance, the mean or median may be used to obtain a point estimate for an estimated parameter, and the posterior standard deviation can be used to describe the uncertainty around that point estimate.

Although it is possible to obtain the posterior distribution analytically for some simple models, the Bayesian estimation of more complex models is usually done with Markov Chain Monte Carlo algorithms, such as Gibb's sampling, which relies on consecutively samples from the conditional distributions of the parameters (rather than directly from their joint distribution, c.f., Casella and George, 1992); when the procedure has converged, one effectively samples from the (joint) posterior distribution. These samples can then be used as an approximation of the underlying posterior distribution, which in turn can be used to obtain point estimates for the parameters. A particularly desirable feature of MCMC procedures is that, based on the samples of the estimated parameters, it is also possible to calculate new statistics and obtain their posterior distribution. For instance, based on the estimated parameters θ, ϕ, and $\sigma_{\epsilon }^{2*}$ for the ARMA(1,1) model, we will calculate the innovation variance $\sigma_{\epsilon }^{2}$ and measurement error variance $\sigma_{\omega }^{2}$ in each sample, such that we obtain posterior distributions for these parameters. In our simulations we use the free open source software JAGS (Plummer, 2003) which employs a Gibb's sampling algorithm, in combination with the R package Rjags (Plummer et al., 2014).

### 3.3. Simulation conditions

Throughout the simulation study, we simulated 1000 data sets per condition according to the AR(1)+WN model specified in Equations (3–5) using R (R Development Core Team, 2012). For all conditions, the mean of the model is fixed to 2. The study consists of three parts. First, we examine the effect of *the proportion of measurement error variance to the total variance*, on parameter recovery. The total variance for the AR(1)+WN is the sum of the variance for an AR(1) model and the measurement error variance: $\sigma_{total}^{2}=\sigma_{\epsilon }^{2}∕\left(1-\phi^{2}\right)+\sigma_{\omega }^{2}$ (c.f., Harvey, 1989; Kim and Nelson, 1999). To vary the proportion of $\sigma_{\omega }^{2}$ to the total variance, ϕ and $\sigma_{\epsilon }^{2}$ are both fixed to 0.5 in this study while the measurement error variance is varied. Specifically, the measurement error variance takes on the values 0, 0.1, 0.2, 0.3, 0.5, 0.7, 1, 2, 4, and 12, which results approximately in the following proportions of measurement error variance to the total variance: 0, 0.13, 0.23, 0.31, 0.43, 0.51, 0.6, 0.75, 0.86, and 0.95.

Second, we examine the effect of *the size of* ϕ on parameter recovery. We vary ϕ over the values −0.75, −0.5, −0.25, 0, 0.25, 0.5, and 0.75. The proportion of measurement error variance to the total variance of the AR(1)+WN process is fixed to 0.3 here, through varying the innovation variances $\sigma_{\epsilon }^{2}$ by approximately 1.2, 1.1, 0.9, 0.5, 0.9, 1.1, and 1.2 respectively.

Third, we examine the effects of *sample size*. In part 1 and 2 of the study we use a sample size 100 repeated measures. We based this number roughly on what one may expect for research in psychology: Typically, what we see in time series applications in psychology is a range of about 60–120 repeated measures per person (e.g., see Nezlek and Gable, 2001; Rovine and Walls, 2006; Madhyastha et al., 2011; Ferrer et al., 2012; Wang et al., 2012; Adolf et al., 2015). However, in preliminary analyses we found difficulties in estimating the model with a small sample size, especially for the frequentist estimation procedure, that pointed to empirical underidentification (we elaborate on this in the next section). Therefore, we varied sample size by 100, 200, and 500. For this part of the study $\sigma_{\epsilon }^{2}$, $\sigma_{\omega }^{2}$, and ϕ were fixed to 0.5, implying a proportion of measurement error variance to the total variance of 0.43.

We judge the performance of each model based on: (a) its bias in the estimates; (b) the absolute error in the estimates; and (c) coverage rates for the 95% confidence or credible intervals. It is not clear whether Bayesian 95% credible intervals should have exactly 95% coverage rates, however, with uninformative priors we would expect this to be the case. Moreover, we consider it informative to see how often the true value lies within the credible interval across multiple samples (e.g., if this occurs very rarely this seems problematic for making inferences).

For the coverage rates of the variances estimated with the frequentist ML procedure, we calculate the confidence intervals based on a χ^2^ distribution with *n* − 1 degrees of freedom as follows: $CI\left(\frac{\left(n-1\right)s^{2}}{\chi_{1-\alpha ∕2}^{2}},\frac{\left(n-1\right)s^{2}}{\chi_{\alpha ∕2}^{2}}\right)$, where *n* is the sample size, and *s*^2^ is the estimated variance.

### 3.4. Expectations

For part 1, we expect that all models will decrease in performance (i.e., more bias and absolute error, lower coverage rates) as the proportion of measurement error variance increases, because an increase in random noise should make it harder to distinguish an (autoregressive) effect. Furthermore, we expect that the decrease in performance will be larger for the AR(1) model than for the ARMA(1,1) and AR(1)+WN model. Specifically, based on Staudenmayer and Buonaccorsi (2005), we expect a bias in the estimates of ϕ in the AR(1) model of approximately 0, −0.07, −0.12, −0.16, −0.21, −0.26, −0.30, −0.38, −0.43, and −0.47, given that the proportions of measurement error variance are 0, 0.13, 0.23, 0.31, 0.43, 0.51, 0.6, 0.75, 0.86, and 0.95.

For part 2, we expect that the AR(1)+WN and ARMA(1,1) models will improve in performance as the value of |ϕ| increases, given that $\sigma_{\omega }^{2}$ and $\sigma_{\epsilon }^{2}$ should be more easily distinguished from each other as |ϕ| approaches 1. We are specifically interested in the performance of the AR(1)+WN model compared to the ARMA(1,1) model when |ϕ| is relatively small. Given that the ARMA(1,1) model is identified regardless of the value of ϕ, we expect the ARMA(1,1) model may converge better, and therefore to perform better when ϕ is relatively close to zero than the AR(1)+WN model, which is no longer identified when ϕ is equal to zero.

For part three, we expect that performance will improve as sample size increases for the ARMA(1,1) model and the AR(1)+WN model, both in the frequentist and Bayesian estimation procedure. Finally, we expect that the Bayesian procedure will perform better than the frequentist state-space procedures for smaller sample sizes, given that both modeling procedures have similar benefits, but the Bayesian estimation procedure is not dependent on large sample asymptotics (Dunson, 2001; Lee and Wagenmakers, 2005).

## 4. Simulation study results

In this section we present the results of the simulation study. As was mentioned before, for a sample size of 100 we found some convergence issues especially for the frequentist ML procedure. Given that convergence is an important precondition for obtaining reasonable parameter estimates, we start by discussing the convergence of the Bayesian models and frequentist models across the different parts of the simulation study. After that, we discuss the parameter recovery performance for each condition specific for each of the three parts of the simulation study. We end with a summarizing conclusion.

### 4.1. Convergence of the bayesian procedures

For the Bayesian procedures we obtained three chains of 40,000 samples each for each replication, half of which was discarded as burn-in. We judged convergence based on the multivariate Gelman-Rubin statistic and autocorrelations for all replications, and we inspected the mixing of the three chains visually a number of replications (c.f., Gelman and Rubin, 1992; Brooks and Gelman, 1998). For the AR(1) model the chains mixed well, the Gelman Rubin statistic was generally equal to one, and the autocorrelations for the parameters decreased exponentially across all conditions.

For the ARMA(1,1) the chains generally mixed well, and the Gelman Rubin statistic was equal to one across all conditions.^3^ The autocorrelations for the parameters decreased slower than for the AR(1) model, and decreased most slowly when the proportion of measurement error variance was higher than 50% or |ϕ| was zero.

For the AR(1)+WN model, overall the chains mixed well and the Gelman Rubin Statistic was equal to one for most replications. For approximately 1–2% of the data sets the Gelman Rubin statistic was larger than 1.1, indicating possible non-convergence, with the exception of the condition where ϕ = 0.75, for which it was 8%. Closer inspection indicated that these problems usually originated and were limited to μ. The percentage of non-convergence is larger for the condition ϕ = 0.75, most likely because when ϕ is strong and positive it is most difficult to estimate μ because observations may tend to linger longer above or below the mean. The autocorrelations for the AR(1)+WN model are higher overall, and slower to decrease than those for the AR(1) and ARMA(1,1) model across all conditions. More measurement error and a closer |ϕ| to zero, was associated with more slowly decreasing autocorrelations.

### 4.2. Convergence of the (frequentist) ML with state-space modeling procedures

For the ML procedure we encountered three types of problems: (1) negative standard errors for the estimated parameters, (2) optim failing to initialize (more rarely), and (3) Heywood cases (negative variances) for the measurement error variance or the innovation variance. The first and second type of problem could usually be resolved by providing alternative starting values and rerunning the model. For a small percentage of data sets, five sets of starting values still did not resolve these issues (for the number of data sets per condition, see Table A1 in Supplementary Materials). These data sets are excluded from the parameter recovery results. When sample size was increased to 200 or 500 repeated measurements, these problems were no longer encountered.

The third type of problem—Heywood cases—was much more prevalent, and could generally not be resolved by providing different starting values. For the AR(1)+WN model, for 10–55% of the replications $\sigma_{\omega }^{2}$, or more rarely $\sigma_{\epsilon }^{2}$, were estimated at the lower bound of zero. For the ARMA(1,1) model, we similarly detected Heywood cases for $\sigma_{\omega }^{2}$ and $\sigma_{\epsilon }^{2}$ (note that $\sigma_{\omega }^{2}$ and $\sigma_{\epsilon }^{2}$ are calculated a posteriori based on the estimated ϕ, θ and $\sigma_{\epsilon }^{2*}$ by means of Equations 8 and 9). In the case that for the AR(1)+WN model $\sigma_{\omega }^{2}$ or $\sigma_{\epsilon }^{2}$ were estimated at the lower bound, usually a Heywood case would also observed for the ARMA(1,1) model for that replication. The proportions of Heywood cases for $\sigma_{\omega }^{2}$ and $\sigma_{\epsilon }^{2}$ across all conditions are reported in Table A1 in the Supplementary Materials.

The number of Heywood cases increased when: (1) ϕ got closer to zero, such that it is harder to discern measurement errors from innovations (2) when there was very little measurement error, such that $\sigma_{\omega }^{2}$ was already close to zero, and (3) There was a lot of measurement error, such that all parameter estimates were uncertain (large standard errors). This indicates issues of empirical identification, and as such we expected these issues to decrease as sample size increases.

The Heywood cases for $\sigma_{\epsilon }^{2}$ and $\sigma_{\omega }^{2}$ decreased as sample size increased—however, the issues were not resolved completely: For *n* = 200 almost 30% of the data sets still returned a Heywood case, and for *n* = 500 almost 13% still returned a Heywood case. Given that for smaller sample sizes (e.g., less than 500), which are much more common in psychological studies, the proportion of replications with Heywood cases was quite large for many conditions, this seems quite problematic. In practice, encountering such a result may lead a researcher to erroneously conclude that there most likely is no considerable measurement error variance, so that a regular AR(1) model should suffice.

In the following sections, where we discuss the parameter recovery results, the data sets with Heywood cases for $\sigma_{\omega }^{2}$ or $\sigma_{\epsilon }^{2}$ are included in the results, because to exclude so many data sets would make a fair comparison to the Bayesian procedure (for which no data sets need to be excluded) problematic. However, the results with these data sets excluded for the ML AR(1)+WN model and ARMA(1,1) model are presented and discussed in Supplementary Materials. Finally note that, in contrast to our expectations, in the ML procedure the ARMA(1,1) model does not seem to converge more easily than the AR(1)+WN model. In general it seems that in order to properly estimate and distinguish the measurement error variance from the innovation variance using ML, quite large sample sizes are required.

### 4.3. Parameter recovery for different proportions of measurement error

**Figure 2:**
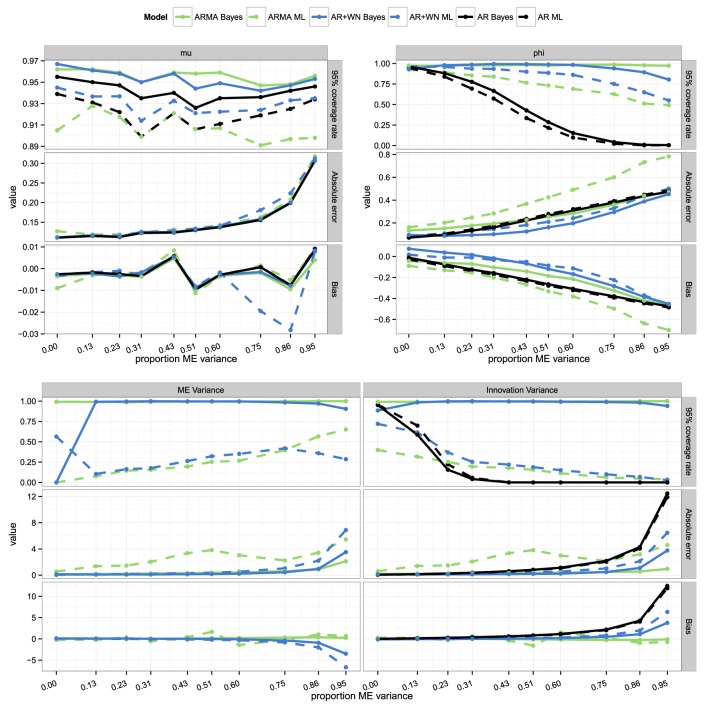
**Coverage rates, absolute errors, and bias for the parameter estimates for the frequentist and Bayesian AR(1), ARMA(1,1), and AR(1)+WN models across different proportions of measurement error variance to the total variance**.

In general, as the proportion of measurement error increases, the estimated parameters become increasingly more biased, the absolute errors become larger, and coverage rates become lower, as expected. In Figure 2 we provide plots of the 95% coverage, absolute errors, and bias for each model, condition, and parameter. As can be seen from this figure, overall, the Bayesian AR(1)+WN model outperforms the other procedures in terms of coverage rates and absolute errors, and for the variance parameters also in terms of bias. The ML state-space AR(1)+WN model performs second-best overall, and performs the best for ϕ in terms of bias. The Bayesian and frequentist AR(1) and ARMA(1,1) models perform relatively poorly in all respects. However, the ARMA(1,1) models result in better coverage rates for ϕ than the AR(1) models, so that an ARMA(1,1) model is still preferred over a simple AR(1) model. Below, we will discuss the results in more detail, per parameter.

For μ, all models perform similarly well in terms of bias and absolute error, as can be seen from the top-left panel of Figure 2. In terms of coverage rates, the Bayesian AR(1) and AR(1)+WN model outperform the other models for μ, most pronouncedly when the proportion of measurement error is high.

For ϕ, the models that perform the best in terms of bias are the ML AR(1)+WN model, followed by the Bayesian AR(1)+WN model (see the top-right panel in Figure 2). The bias for ϕ in both AR(1) models is in line with our expectations, increasing from approximately 0 to −0.5 as measurement error increases. As can be seen from the top-right panel of Figure 2, in terms of absolute error for ϕ, the Bayesian AR(1)+WN model performs the best, followed by the ML AR(1)+WN model. The top-right panel of Figure 2 shows that the coverage rates for ϕ based on the 95% CI's for the Bayesian ARMA(1,1) model are consistently higher than those for the other models, however, this is a result of having wider credible intervals, rather than a result of more precise estimates for ϕ. The coverage rates for the Bayesian AR(1)+WN model are most stable across the different proportions of measurement error variance. The coverage rates for this Bayesian model are generally higher than 0.95^4^, only dropping below 0.95 when 75% or more of the total variance is measurement error variance. In comparison, the ML AR(1)+WN model starts with a coverage rate of approximately 0.95 for ϕ when measurement error is absent, and the coverage decreases as measurement error increases (with a lowest coverage of 0.55 when 95% of the variance is due to measurement error). The ML ARMA(1,1) model and the Bayesian and ML AR(1) models perform the worst, as can be seen from Figure 2. Note that for the AR(1) models, the coverage rates for ϕ are already below 90% when the proportion of measurement error variance is as little as 0.13.

In the bottom panel of Figure 2 the results for $\sigma_{\omega }^{2}$ and $\sigma_{\epsilon }^{2}$ are displayed. When the proportion of error variance is larger than about 0.3, the Bayesian AR(1)+WN model starts to outperform the ML AR(1)+WN model in terms of bias for $\sigma_{\omega }^{2}$ and $\sigma_{\epsilon }^{2}$. Further, it can be seen from Figure 2 that for the AR(1)+WN models, when the proportion of measurement error is small, the measurement error variance is slightly overestimated, while when the proportion of measurement error is large, the measurement error variance is underestimated. The coverage rates are the highest for the Bayesian AR(1)+WN and ARMA(1,1) model. Note that for the ARMA(1,1) model $\sigma_{\omega }^{2}$ and $\sigma_{\epsilon }^{2}$ are calculated based on the estimated ARMA(1,1) parameters. For the Bayesian model this was done in each Gibbs sample by means of Equations (8) and (9), resulting in a posterior distribution for $\sigma_{\omega }^{2}$ and $\sigma_{\epsilon }^{2}$. However, depending on the specific values of the ARMA(1,1) parameters in each Gibbs sample, $\sigma_{\omega }^{2}$ and $\sigma_{\epsilon }^{2}$ may become quite large or even negative. As a result, the posterior standard deviations and credible intervals for $\sigma_{\omega }^{2}$ and $\sigma_{\epsilon }^{2}$ in the Bayesian ARMA(1,1) model can be quite large, including negative and large positive values. The confidence intervals for the variances parameters in frequentist procedures are consistently too narrow, which results in low coverage rates, as can be seen from the bottom panel of Figure 2. As such, for the two variances, the Bayesian AR(1)+WN model performs best in terms of coverage rates, followed by the Bayesian ARMA(1,1) model (which has higher coverage rates, but much wider intervals), and the ML AR(1)+WN model. The same pattern holds for the absolute errors as can be seen in Figure 2.

### 4.4. Parameter recovery for different values of ϕ

**Figure 3:**
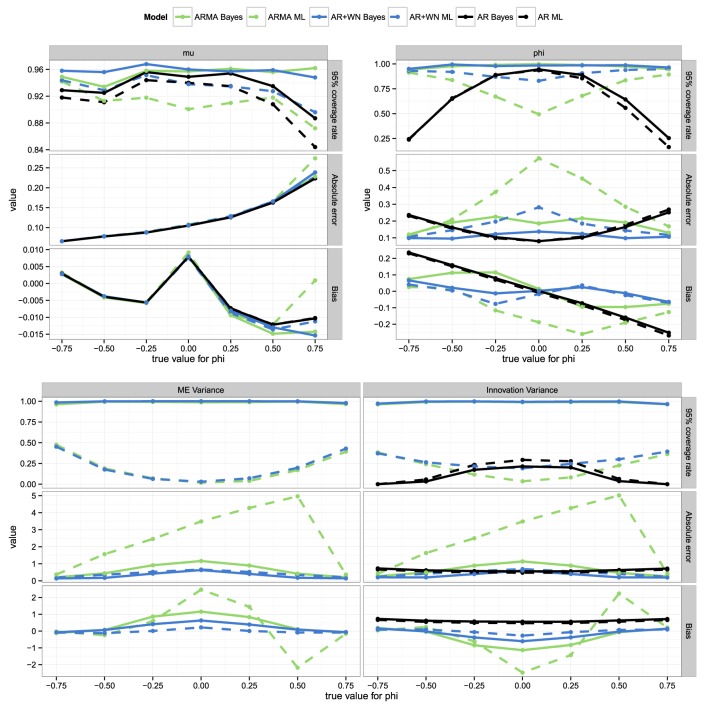
**Coverage rates, absolute errors, and bias for the parameter estimates for the frequentist and Bayesian AR(1), ARMA(1,1), and AR(1)+WN models across different values for ϕ**.

For this part of the study, the value of ϕ was varied from −0.75 to −0.5, −0.25, 0, 0.25, 0.5, and 0.75. As can be seen from the top-left panel of Figure 3, for μ all the models perform very similarly in terms of bias, absolute errors, and coverage rates. The absolute errors and bias increase as ϕ becomes larger, because when ϕ is strong and positive, observations may tend to linger longer above or below the mean than when ϕ is weak or negative, making it harder to estimate μ.

As can be seen from the top-right and bottom panel of Figure 3, the results for ϕ and the variance parameters are symmetric for negative and positive values of ϕ (or mirrored in the case of bias). As such, we will discuss these results in terms of |ϕ|. For the parameters ϕ, $\sigma_{\epsilon }^{2}$ and $\sigma_{\omega }^{2}$, performance increases as |ϕ| increases, except the AR(1) models, for which it is the opposite. Overall, the Bayesian AR(1)+WN performs best, followed by respectively the ML AR(1)+WN model, the Bayesian ARMA(1,1) model, and the ML ARMA(1,1) model. The performance of the latter three models decreases considerably more as |ϕ| decreases than that of the Bayesian AR(1)+WN model, as can be seen from Figure 3.^5^ For the two variances, the ML AR(1)+WN model outperforms the Bayesian model in terms of bias. Finally, we find that when |ϕ| is relatively close to one, the measurement error variance is underestimated, however, when |ϕ| is relatively small, the measurement error variance was actually overestimated, as can be seen from the bottom panel of Figure 3.

### 4.5. Parameter recovery for different sample sizes

**Figure 4:**
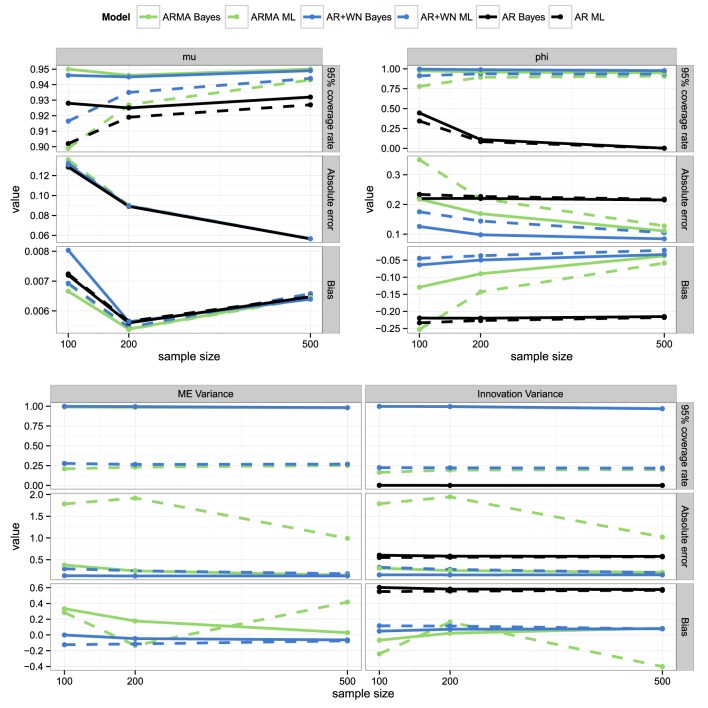
**Coverage rates, absolute errors, and bias for the parameter estimates for the frequentist and Bayesian AR(1), ARMA(1,1), and AR(1)+WN models across sample sizes**.

For this part of the simulation study, the sample size was varied from 100 to 200 and 500. As shown in Figure 4, as sample size increases, parameter recovery improves: Bias and absolute errors decrease, while coverage rates become closer to 0.95. We Further, the ML AR(1)+WN results become more similar to those of the Bayesian AR(1)+WN model as sample size increases, although the Bayesian model still outperforms the ML model in terms of absolute error and coverage: The Bayesian procedure results in higher coverage rates, but less wide intervals, that is, in more precise estimates than the ML procedure for ϕ. Note that the performance of the ML and Bayesian ARMA(1,1) models only near the performance of the AR(1)+WN models as sample size has increased to 500 observations.

### 4.6. Conclusion

Overall, the Bayesian AR(1)+WN model performs better than the other five procedures we considered. We expected that the ARMA(1,1) models may outperform the AR(1)+WN models in parameter recovery, because we expected this model to have less trouble with identification and convergence. Interestingly, although the Bayesian ARMA(1,1) model seems to converge more easily than the Bayesian AR(1)+WN model, the AR(1)+WN model still outperforms the ARMA(1,1) model in terms of parameter recovery, even when ϕ is close or equal to zero. The ML AR(1)+WN model and ARMA(1,1) models are both unstable for small sample sizes (*n* = 100), frequently resulting in Heywood cases for the innovation and measurement error variances. However, the ML AR(1)+WN model still performs relatively well for estimating ϕ compared to the AR(1) models. For a smaller sample size of 100 observations the Bayesian procedure outperforms the frequentist ML procedure. When sample sizes are larger, the discrepancies between the Bayesian and frequentist AR(1)+WN model decrease, although the confidence intervals for the variance parameters in the frequentist procedures are consistently too narrow. As expected, the AR(1) models severely underestimate |ϕ|, which is reflected in large bias and absolute errors, and low coverage rates. Finally, we note that although the AR(1)+WN models perform considerably better than the AR(1) models, some bias in ϕ still remains, because the innovations and measurement errors cannot be perfectly discerned from each other. Generally, the more measurement error and the lower |ϕ|, the more the estimate of |ϕ| will be biased, even when measurement error is taken into account by the model.

## 5. Empirical application on mood data

To further illustrate the AR(1), ARMA(1,1), and AR(1)+WN model discussed above, we make use of time series data that was collected from female first year social science students at Utrecht University in 2007. Eleven women kept a daily electronic diary for approximately 3 months (across participants the minimum was 90 observations, the maximum 107 observations), in which they filled out how they felt that day on a scale from 1 to 100—1 meaning worst ever, and 100 meaning best ever. Three of the eleven women were excluded from the current study because of non-compliance, issues with the electronic devices, and one woman had very little variation in her scores. For the remaining women the average number of missing observations was approximately nine. Values for these missing observations will be automatically imputed as part of the estimation procedure, based on the specified model.

We are interested in finding out to what extent current mood influences mood the following day. As such, we are interested in fitting an AR(1) model, and specifically in the AR effect reflected in parameter ϕ. However, the mood of each person is not likely to be perfectly measured. For instance, it is possible that participants accidentally tapped the wrong score when using the electronic diary stylus to fill in the questionnaire. Furthermore, the participants evaluate their mood for the day on average, such that momentary influences around the time of filling out the diary may have colored their evaluation of the whole day (i.e., a form of retrospective bias). In fact, anything that is not explicitly measured and modeled, and of which the influence does not carry-over to the next day, can be considered measurement error. As such, it seems likely that there is at least some measurement error present in the data. Therefore, we fit the AR(1)+WN model to take this measurement error into account, and for illustrative purposes compare it to an ARMA(1,1) model, and an AR(1) model (which disregards measurement error). The data and codes for running the analyses are included in the Supplementary Materials. We make use of a Bayesian modeling procedure, given that the results from our simulation study indicate that the parameter recovery performance of the Bayesian procedure is better and more stable for this number of repeated measures. The priors we use for the models are aimed to be uninformative, specifically: A *uniform*(0, 500) prior distribution for all variance parameters, a *uniform*(−1, 1) prior distribution for ϕ and θ, and a *normal*(0, 0.001) prior distribution for μ (specified with a precision rather than a variance).

We evaluated the convergence of the AR(1), ARMA(1,1), and AR(1)+WN model by visually inspecting the mixing of the three chains, the Gelman Rubin statistic, and the autocorrelations. For the AR(1) and AR(1)+WN model the chains mixed well, the Gelman Rubin statistic was approximately equal to one, and the autocorrelations for the parameters decreased within 50–100 lags across all participants. For the ARMA(1,1) model this was the case, except for participants 3 and 8.^6^ We included the ARMA(1,1) estimates for these participants in Table 1, but these should be interpreted with caution.

**Table 1:** **Parameter estimates for the AR(1), ARMA(1,1), and AR+WN model for the mood of eight women, estimated with Bayesian software**.

**Pp**	**Model**	**μ (95%CI)**	**ϕ (95%CI)**	**$\sigma_{\epsilon }^{2}$ (95%CI)**	**$\sigma_{\omega }^{2}$ (95%CI)**	$\sigma_{\epsilon }^{2*}$ (95%CI)	**θ (95%CI)**
1	AR1	75 (72, 79)	0.08 (−0.17, 0.32)	166 (122, 235)	–	–	–
	ARMA	76 (72, 81)	0.53 (−0.32, 0.90)	21.34 (− 91, 180)	125 (− 6, 278)	160 (117, 227)	−0.41 (−0.81, 0.29)
	ARWN	76 (72, 79)	0.39 (−0.23, 0.77)	42 (3, 160)	112 (16, 193)	–	–
2	AR1	63 (59, 68)	0.36 (0.13, 0.57)	188 (141, 256)	–	–	–
	ARMA	63 (58, 69)	0.48 (−0.21, 0.97)	103 (−740, 1087)	69 (−870, 960)	189 (142, 257)	−0.13 (−0.64, 0.49)
	ARWN	63 (58, 68)	0.52 (0.15, 0.84)	101 (20, 208)	77 (7, 184)	–	–
3	AR1	63 (61, 66)	0.21 (0, 0.42)	108 (81, 148)	–	–	–
	ARMA	64 (61, 66)	0.02 (−0.72, 0.81)	−1 (−288, 251)	109 (−134, 418)	105 (79, 144)	0.19 (−0.64, 0.95)
	ARWN	64 (61, 67)	0.40 (−0.01, 0.82)	38 (4, 112)	64 (6, 118)	–	–
4	AR1	56 (53, 58)	0.21 (0.01, 0.42)	103 (78, 141)	–	–	–
	ARMA	54 (40, 59)	0.85 (0.35, 0.99)	7 (1, 47)	75 (44, 112)	95 (71, 130)	−0.68 (−0.87,−0.14)
	ARWN	55 (49, 59)	0.69 (0.07, 0.97)	19 (2, 88)	70 (17, 111)	–	–
5	AR1	69 (64, 75)	0.48 (0.28, 0.67)	174 (131, 239)	–	–	–
	ARMA	69 (62, 77)	0.67 (0.20, 0.92)	86 (24, 348)	61 (−139, 143)	173 (130, 237)	−0.26 (−0.58, 0.24)
	ARWN	69 (62, 77)	0.67 (0.37, 0.91)	90 (27, 190)	66 (6, 140)	–	–
6	AR1	73 (71, 74)	0.27 (0.07, 0.46)	31 (24, 42)	–	–	–
	ARMA	73 (71, 74)	0.18 (−0.43, 0.66)	22 (−305, 349)	8 (−314, 339)	31 (24, 42)	0.09 (−0.45, 0.61)
	ARWN	73 (71, 74)	0.33 (0.01, 0.62)	21 (4, 35)	10 (0.51, 30)	–	–
7	AR1	71 (69, 73)	0.08 (−0.13, 0.28)	105 (79, 144)	–	–	–
	ARMA	71 (65, 75)	0.48 (−0.77, 0.99)	7 (−132, 175)	87 (−63, 248)	104 (78, 142)	−0.36 (−0.90, 0.77)
	ARWN	71 (68, 74)	0.26 (−0.57, 0.92)	23 (1, 101)	76 (8, 123)	–	–
8	AR1	73 (71, 74)	0.03 (−0.18, 0.24)	59 (44, 80)	–	–	–
	ARMA	73 (71, 74)	−0.22 (−0.81, 0.84)	−5 (−131, 102)	67 (−41, 197)	57 (43, 78)	0.31 (−0.98, 0.95)
	ARWN	73 (71, 74)	−0.03 (−0.65, 0.51)	16 (0.35, 61)	42 (2, 70)	–	–

Note that the negative values for in the credible interval for $\sigma_{\epsilon }^{2}$ and $\sigma_{\omega }^{2}$ for the ARMA(1,1) models result, because they are calculated a posterior based on the samples for ϕ, θ, and $\sigma_{\epsilon }^{2*}$ based on Equations (8) and (9): It is possible that for certain combinations of these parameters $\sigma_{\epsilon }^{2}$ and $\sigma_{\omega }^{2}$ become negative. For participants 3 and 8 the ARMA(1,1) model did not converge properly, so that these results should be interpreted with caution.

The parameter estimates of the mean μ, AR parameter ϕ, innovation variance $\sigma_{\epsilon }^{2}$, measurement error variance $\sigma_{\omega }^{2}$, and moving average parameter θ for each person are presented in Table 1. For most of the eight individuals, the baseline mood is estimated to be around 60–70, which indicates that on average they are in moderately good spirits. Further, we see that across models and persons, the AR parameters are either estimated to be positive, or nearly zero. Participant 8 has an AR effect near zero in both the AR(1) model and the AR(1)+WN model, so that for her, everyday seems to be a “new day”: How she felt the previous day does not predict her overall mood today. On the other hand, for participants 2, 4, 5, and 6, the credible intervals for ϕ include only positive values across models: how they feel today depends in part on how they felt yesterday. For the remaining individuals, 1, 3, and 7, the point estimates for ϕ are also positive, however, the credible intervals including negative and positive values for ϕ.

When we compare the results for the AR(1) model and the AR(1)+WN model, we find that for all participants except participant 8, the AR parameter is estimated to be higher in the AR(1)+WN model: Because the AR(1) model does not take measurement error into account, the AR parameter is estimated to be lower than for the AR(1)+WN model. The extent to which the estimate for ϕ differs across the AR(1) and AR(1)+WN model, differs from person to person. The larger the estimated measurement error variance relative to the total variance, the larger the difference between the estimated ϕ in the AR(1) and AR(1)+WN model. For instance, for participants 4 and 6 their estimates of ϕ in the AR(1) model are quite similar to each other (i.e., 0.21 and 0.27), but because the measurement error variance for participant 4 is estimated to be much larger than that for participant 6 (i.e., 70 vs. 10), her ϕ in the AR(1)+WN model ϕ is also estimated to be larger (i.e., 0.69 vs. 0.33).

Note that the ARMA(1,1) and AR(1)+WN model should not necessarily give the same results: Although the AR(1)+WN model is equivalent to the ARMA(1,1) model, the reverse is not the case. In other words, it is possible that the ARMA(1,1) model captures a different pattern of variation in the data than the AR(1)+WN model, giving different results. However, when we compare the results for the ARMA(1,1) and AR(1)+WN model, we do find fairly similar results for most of the participants (with exception of participants 3 and 8, who had convergence issues for the ARMA(1,1) model), especially for participants 2 and 5. However, a clearly notable difference is that the ARMA(1,1) model has less precise estimates than the AR(1)+WN model, as can be seen from the relatively wide credible intervals for the ϕ parameters in Table 1.

Finally, we note that when we calculate the estimated proportion of measurement error variance relative to the total variance based on the AR(1)+WN model for each participant, we find a range of 0.34–0.50 (i.e., 0.36, 0.47, 0.48, 0.50, 0.46, 0.42, 0.46, and 0.34 respectively). This implies that across these eight women, between one third to half of the observed variance is estimated to be due to measurement error.

## 6. Discussion

In this paper we demonstrate that it is important to take measurement error into account in AR modeling. We illustrated the consequences of disregarding measurement error present in the data both in a simulation study, and an empirical example based on a replicated time series design. Further, we compared the parameter recovery performance for the Bayesian and frequentist AR(1)+WN and ARMA(1,1) models that account for measurement error. Ignoring measurement error present in the data is known to result in biased estimates toward zero of the AR effects in AR(1) models, with the extent of the bias depending on the proportion of measurement error variance and the size of ϕ (Staudenmayer and Buonaccorsi, 2005). Our simulations also demonstrated this bias, and showed large absolute errors and importantly, very poor coverage rates for the AR effect when measurement error is disregarded, regardless of sample size. For research in psychology, for which it is very difficult or perhaps impossible to measure error-free, it seems imperative to consider this potentially large source of variance in our (AR) time series models. In our empirical application for instance, between one third to half of the variance in the data is estimated to be due to measurement error.

Comparing the parameter recovery for the models that incorporate measurement error—the Bayesian and ML ARMA(1,1) model and AR(1)+WN model—revealed that the Bayesian AR(1)+WN model performed best in terms of parameter recovery. It proved relatively tricky to properly estimate the ML ARMA(1,1) and AR(1)+WN model, even for larger sample sizes of 500 repeated measures: These models are prone to Heywood cases in the measurement error variance and to a lesser extent in the innovation variance. This was especially common (up to 55% of the replications) when AR effect was closer to zero, or the amount of measurement error was large. In practice, hitting such a lower bound for the measurement error variance may erroneously suggest to researchers that the model is overly complex, and that there is no notable measurement error present in the data, which is problematic.

Note that while 100 observations may be small for estimation purposes, it is quite a large number of repeated measures to collect in practice. In psychological research using intensive longitudinal data, we usually see no more than about 120 observations per person (to illustrate, 120 observations would arise from about 4 months of daily measurements, or for more intense 2 weeks regime, measuring someone 9 times a day). Fortunately, the Bayesian AR(1)+WN model provides a good option even for such small sample sizes. Still, the models that incorporate measurement error need more observations to give as precise estimates as the basic AR(1) model, which has relatively small credible/confidence intervals (although this is precision around a wrong estimate when there actually is measurement error present in the data). Therefore, it seems good practice to take potential measurement error into account in the design of the study, thus collecting more repeated measures in order to compensate for any potential measurement error that has to be filtered out later. Expectedly, and as is shown in the simulation study, this becomes especially important when the proportion of measurement error variance is relatively large, or when the AR effects are (expected to be) relatively small. One option to improve the estimates may be to use (weakly) informative prior specifications based on previous research, or expert knowledge. However, prior information on the model parameters may currently prove difficult to obtain, given that studies that estimate measurement error or take it into account are very rare, and that the model parameters differ from person to person, and from variable to variable. Another option could be to extend the AR+WN model to a multilevel model, assuming a common distribution for the parameters of multiple individuals, and allowing the model parameters to vary across persons. By making use of this hierarchical structure that can take similarities between persons into account, a relatively low number of time points may be compensated for to some extent by a large number of participants, which may be easier to obtain (for examples of the multilevel AR(1) model, see Rovine and Walls, 2006; Lodewyckx et al., 2011; De Haan-Rietdijk et al., 2014).

The reader may wonder how one may determine if there is, or isn't, measurement error present in the data. One way to do this is to use information criteria to compare the AR(1) model with the ARMA(1,1) or AR(1)+WN model. Although a thorough study of model selection is beyond the scope of the current paper, we provide some preliminary evaluations of the model selection performance of the AIC, BIC, and DIC, in Supplementary Materials. We find that these criteria frequently incorrectly selects the simpler AR(1) model over the (true) AR(1)+WN model and ARMA(1,1) model, so that these criteria seem inappropriate for selecting between the AR(1) and the ARMA(1,1) model or the AR(1)+WN model in this context. Selecting between an AR(1)+WN model and an ARMA(1,1) model will also be problematic using standard information criteria, because the AR(1)+WN model may be considered a restricted (simpler) version of the ARMA(1,1) model (see Equation 8), while they have the same number of parameters, and thus the same penalty for complexity for many fit criteria. In that sense, when they have equal fit, the AR(1)+WN model may be preferred because it is the simpler model, but if this is not the case, it becomes more complicated to choose between the two. Directions for future research therefore are to establish information criteria for selecting between the AR(1)+WN model and the AR(1) and ARMA(1,1) model, perhaps using information criteria or Bayes factors developed for restricted parameters (c.f., Dudley and Haughton, 1997; Klugkist and Hoijtink, 2007; Kuiper et al., 2012). Although model selection using information criteria may prove complicated, it is important to note that the estimates for ϕ in the AR(1)+WN models seem to be reasonably accurate, even when there is no measurement error present in the data. Combined with the intuition that most psychological measurements will contain at least some measurement error, fitting the model that incorporates measurement error seems a relatively “safe bet.”

Another interesting topic for future work is how measurement error affects estimates of the effects variables have on each other over time, that is, the cross-lagged effects. This may be especially relevant for individual network models of psychological processes (Schmittmann et al., 2013). For example, in a network model for an individual diagnosed with a depressive disorder, the depression symptoms constitute the nodes in the network, and the AR and cross-lagged effects between the symptoms constitute the connections in this network (Borsboom and Cramer, 2013; Bringmann et al., 2013). It would be interesting to investigate to what extent measurement error in each variable affects the resulting network.

Finally, while incorporating measurement error into time series models is likely to decrease distortions as a result of ignoring measurement error to the parameter estimates, we emphasize that it is not a cure-all. Even in the models that incorporate measurement errors, the AR parameters may be slightly under- or over-estimated, because measurement error variance and innovation variance are not completely discernible from each other. The more measurement error present in the data, the more difficult it will be to pick up any effects. Therefore, there is still a strong argument for preventing measurement errors in the first place. One option to potentially improve the measurements is to use multiple indicators to measure the relevant construct. However, in a intensive longitudinal data setting, using multiple items for each variable would strongly increase the burden on the participant, who would have to repeatedly fill out all these questions. What remains are classical ways of preventing measurement error: Improving the respective measurement instruments, the circumstances under which participants are measured, and explicitly measuring and modeling potential sources of measurement error. Still, any remaining measurement error that could not be prevented, should be taken into account in the respective model. That is, prevention is better than cure—but a cure is better than ignoring the issue.

### Conflict of interest statement

The authors declare that the research was conducted in the absence of any commercial or financial relationships that could be construed as a potential conflict of interest.

## Footnotes

^1^Other exceptions are of course dynamic factor models, and other latent variable models in which the measurement structure for multiple items is explicitly modeled. Here we focus on applications in which each construct is measured with one variable.

^2^We add the ^*^ to ϵ, to distinguish the innovations for the ARMA(1,1,) model from the innovations of the AR(1)+WN model.

^3^By visually inspecting the chains for μ in the ARMA(1,1) model, we found some extreme values for some of the Gibb's samples (visible as large “spikes” in the chains). To limit these extreme values we adjusted the normal prior for μ to have a smaller variance (10), however this did not resolve the issue completely. As a result, the posterior standard deviation for μ was very large, however, the effects on the point estimates and credible intervals seem limited when we compare these results for μ to those of the other models.

^4^While it may seem undesirable that the Bayesian model has “too high” coverage rates, indicating too large credible intervals or exaggerated uncertainty about the estimated parameters, it is important to note that compared to the ML model, the Bayesian estimates actually have smaller posterior standard deviations than the ML standard errors for ϕ.

^5^The diverging patterns in the bias and absolute errors for the ML ARMA(1,1) model is a result of the Heywood cases discussed in Section 4.2; when the Heywood cases are removed the pattern is similar to the patterns of the other models, as can be seen in Figures B1,B2 in Supplementary Materials.

^6^For participants 3 and 8 we found that the estimates for ϕ and θ in the ARMA(1,1) model were very dispersed, varying across the entire range of −1 to 1, switching from negative to positive values. A density plot of their samples revealed a bimodal distribution for ϕ and θ (with one peak around negative values, and one for positive values): This seems to be some form of label switching, which is indicative of (empirical) under–identification of the ARMA(1,1) model for these two participants.
