# Supplementary material for: Incorporating measurement error in n = 1 psychological autoregressive modeling
Source: Front Psychol. 2015 Jul 28;6:1038. doi: 10.3389/fpsyg.2015.01038 (PMC4516825; doi:10.3389/fpsyg.2015.01038)
Supplement: Supplementary file 2 [file DataSheet2.PDF]

Appendix A

Heywood Cases

In Table A1 we provide the proportions of data sets for which the ML AR(1)+WN and ARMA(1,1) procedure failed, and the proportion of data sets for which σ_ω and σ_ϵ were estimated at the lower bound, or to be negative (a Heywood case). In the main text we present results were the data sets for which the procedure failed (Prop failed in Table A1) are excluded for the ML AR(1)+WN and ARMA(1,1) model (not for the remaining models), but data sets with Heywood cases are included. In Figure A1, A2 and A3 we provide results with both the data sets for which the procedure failed, and the data sets with Heywood cases are excluded for the ML AR(1)+WN and ARMA(1,1) model (not for the remaining models). As can be seen from these figures, the results for the frequentist AR(1)+WN and ARMA(1,1) are more similar to the results of the Bayesian procedures. The Bayesian AR(1)+WN model overall outperforms the remaining Bayesian and frequentist models.

Table A1

Proportion of data sets for which the state space AR+WN and ARMA models would not initialize or had negative standard errors (Prop failed), had Heywood cases or hit the lower bound in the estimates of σ_ϵ (Prop Heywood or lower bound σ_ϵ) and σ_ω (Prop Heywood or lower bound σ_ω), across different proportions of measurement error, different values for ϕ, and different sample sizes.

σ^2_ϵ:	0	.1	.2	.3	.5	.7	1	2	4	12
**AR+WN**										
Prop failed	.041	.012	.009	.011	.023	.030	.060	.084	.183	.256
Prop lower bound σ_ω	.544	.456	.361	.370	.324	.288	.296	.358	.399	.355
Prop lower bound σ_ϵ	.002	.003	.006	.005	.020	.024	.038	.065	.075	.087
**ARMA**										
Prop Failed	.179	.030	.013	.005	.020	.040	.054	.088	.106	.112
Prop Heywood σ_ω	.547	.456	.361	.372	.324	.288	.296	.358	.399	.355
Prop Heywood σ_ϵ	.002	.003	.007	.005	.020	.024	.039	.066	.076	.088
ϕ:	-0.75	-0.5	-0.25	0	0.25	0.5	0.75			
**AR+WN**										
Prop failed	.002	.024	.045	.122	.066	.011	.004			
Prop lower bound σ_ω	.089	.362	.479	.430	.471	.321	.104			
Prop lower bound σ_ϵ	0	.002	.035	.115	.051	.003	.001			
**ARMA**										
Prop failed	.002	.008	.062	.104	.042	.006	.001			
Prop Heywood σ_ω	.070	.229	.140	.049	.164	.263	.131			
Prop Heywood σ_ϵ	.001	.066	.266	.544	.457	.166	.017			
N:	100	200	500							
**AR+WN**										
Prop failed	.031	0	0							
Prop lower bound σ_ω	.293	.219	.102							
Prop lower bound σ_ϵ	.020	0	0							
**ARMA**										
Prop failed	.016	.005	0							
Prop Heywood σ_ω	.218	.218	.112							
Prop Heywood σ_ϵ	.228	.084	.016							

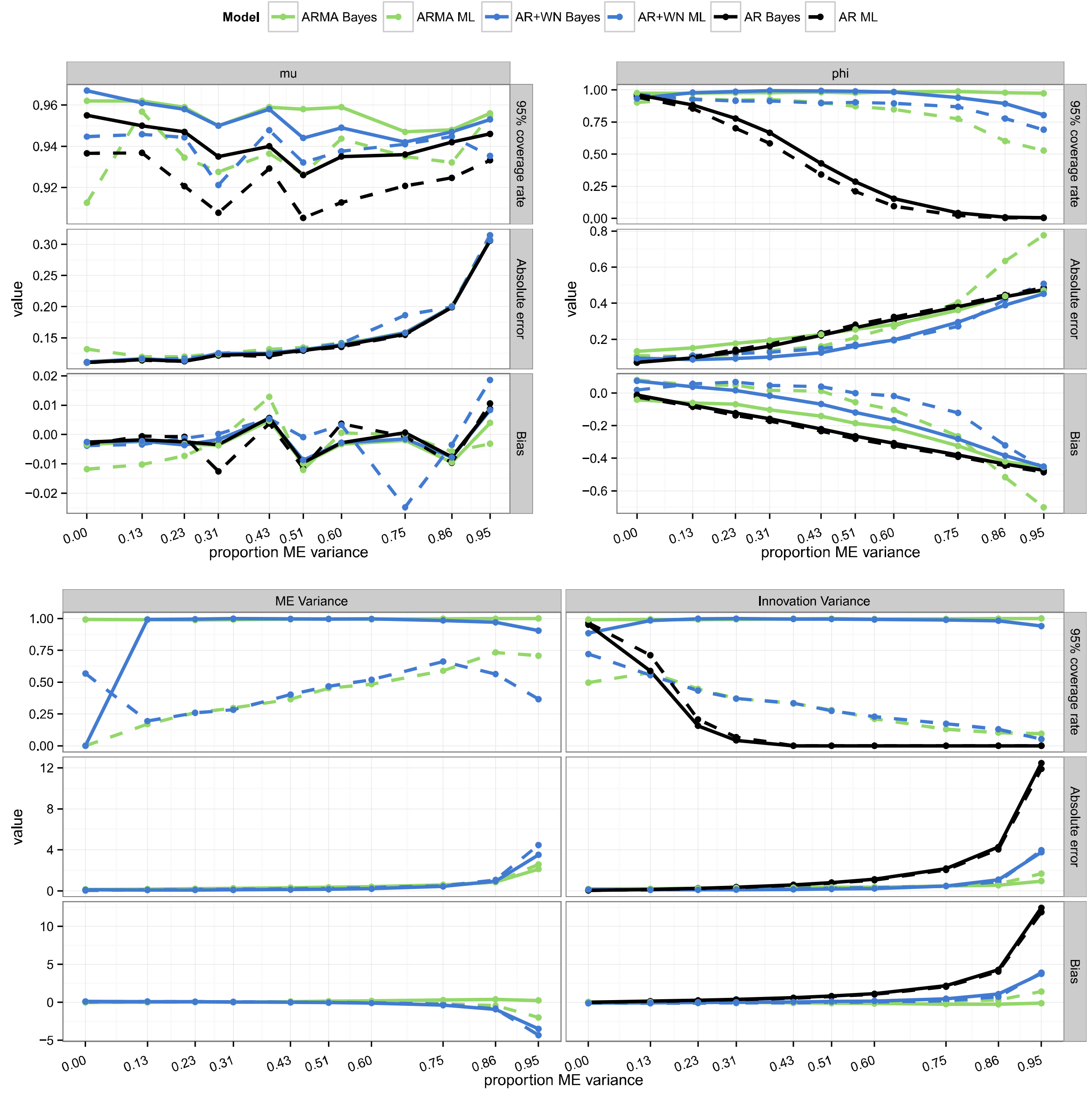

Figure A1. Coverage rates, bias, and absolute errors for the parameter estimates for the frequentist State-space and Bayesian, AR(1), ARMA(1,1), and AR(1)+WN models across different proportions of measurement error variance to the total variance. Data sets with Heywood cases for the frequentist ARMA(1,1) and AR(1)+WN models are excluded here.

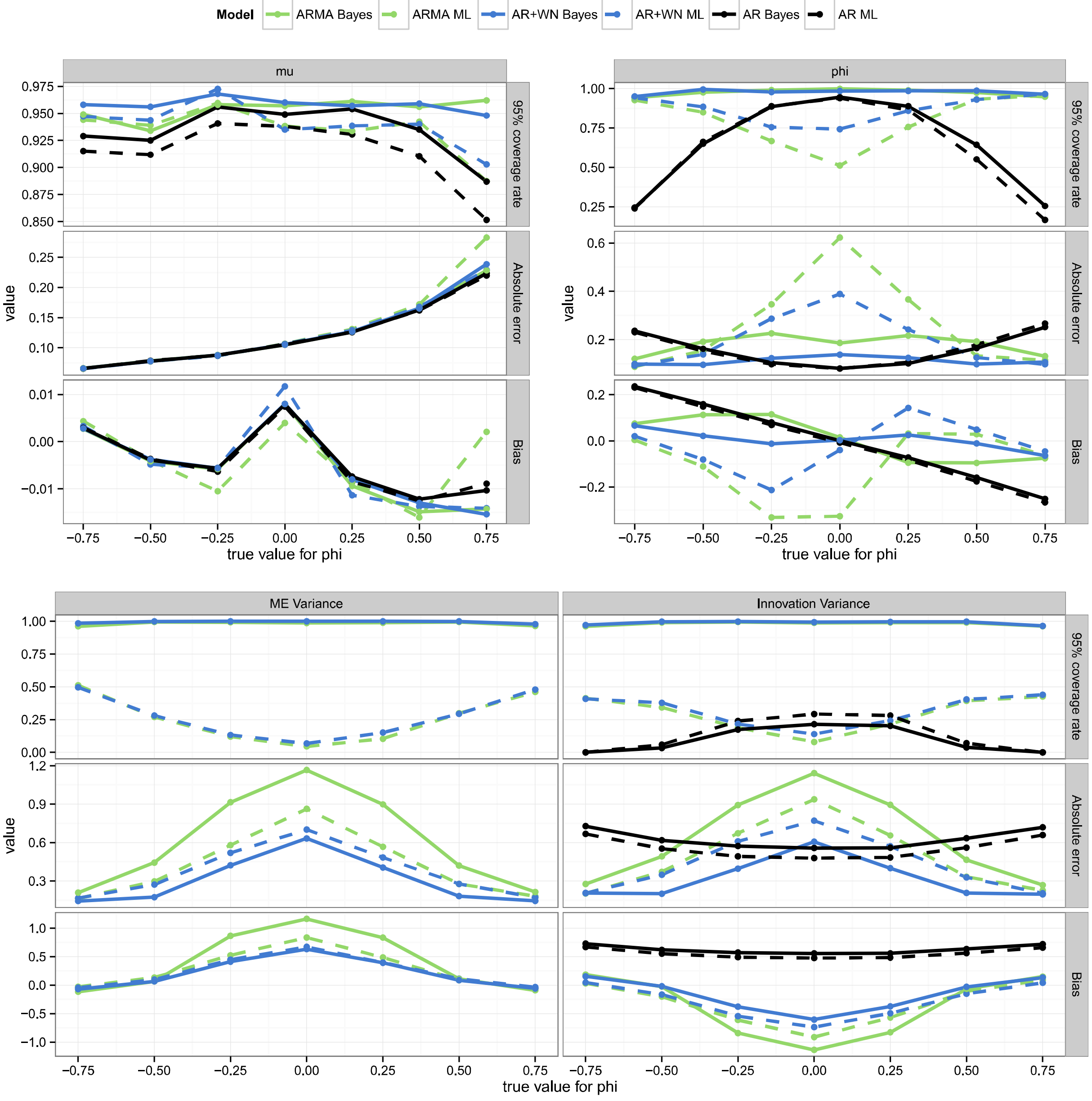

Figure A2. Coverage rates, bias, and absolute errors of the parameter estimates for the frequentist ML State-space and Bayesian AR(1), ARMA(1,1), and AR(1)+WN models across different values for ϕ. Data sets with Heywood cases for the frequentist ARMA(1,1) and AR(1)+WN models are excluded here.

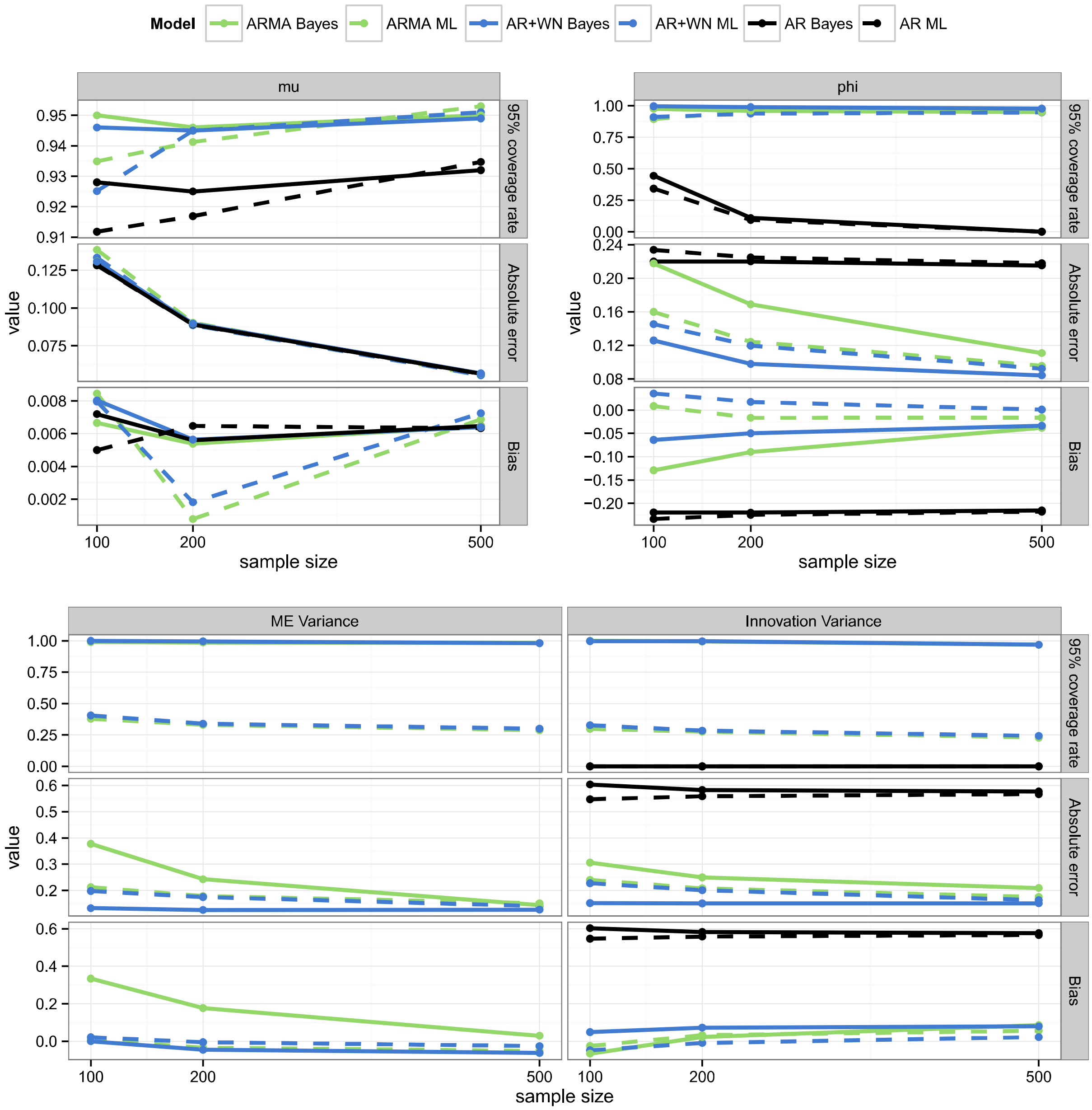

Figure A3. Coverage rates, bias and absolute errors of the parameter estimates for the frequentist ML and Bayesian AR(1), ARMA(1,1), and AR(1)+WN models across different values for ϕ. Data sets with Heywood cases for the frequentist ARMA(1,1) and AR(1)+WN models are excluded here.

Appendix B

Information Criteria Results

While model selection is beyond the scope of this work, we provide some preliminary evaluations here of the model selection performance of the AIC and BIC for the frequentist estimation procedures, and the DIC for the Bayesian estimation procedures. In Figure B1 the average information criteria, as well as the proportion of the 1000 replications for each of the information criteria that the ARMA model was selected over the AR(1) model, and the AR(1)+WN model was selected over the AR(1) model are presented. Based on the AIC, BIC, and DIC, the AR(1) model is selected in favor of the AR(1)+WN and ARMA model for the large majority of replications, even while the latter are the true models. Although the rate the right model is selected improves as sample size increase, for 500 observations the percentage of data sets for which the AR(1)+WN model is correctly selected is still only 50% for the AIC, 40% for the BIC, and 32% for the DIC. As such, the AIC, BIC and DIC are not appropriate for selecting between an AR(1) model and an AR(1)+WN model. The reason for this may be that the measurement error variance and innovation variance are not completely distinct from each other - this depends on the value of ϕ, the higher $|\phi|$ the better the can be distinguished from each other. This is supported by the results presented in the middle panels of Figure C1, which show that as $|\phi|$ increases the proportion of correctly selected models increases.

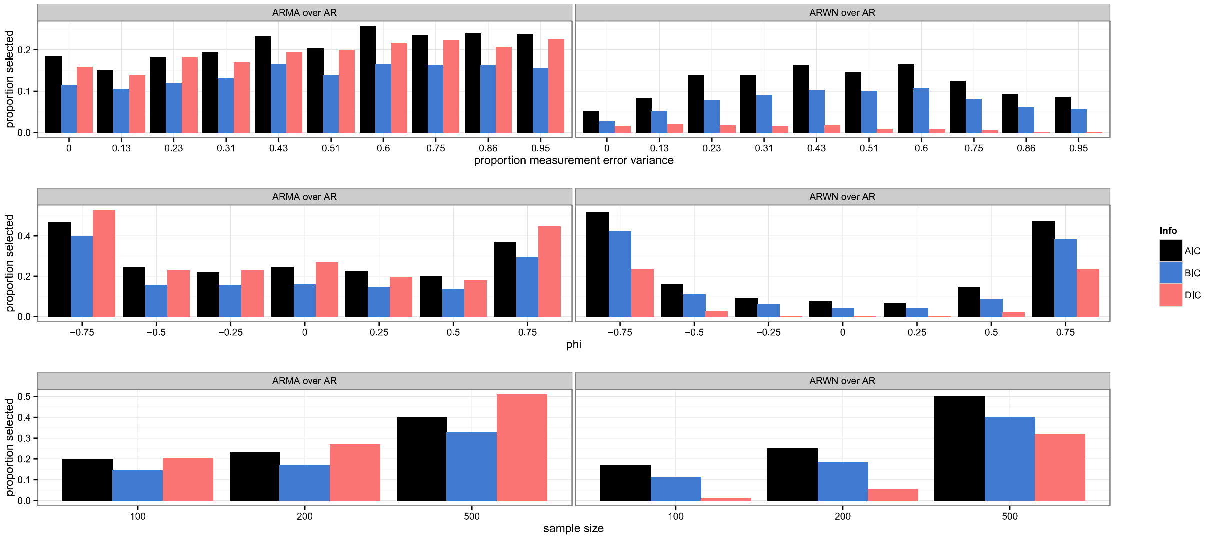

Figure B1. Plots of the proportion the ARMA(1,1) (left panels) and the AR(1)+WN (right panels) that are selected over the AR(1) model per simulation condition, based on the AIC and BIC for the frequentist procedures, and the DIC for the Bayesian procedures.
